# μ-Acetato-bis­(μ-2-{[(3-chloro-2-hydroxy­prop­yl)(2-pyridylmeth­yl)amino]meth­yl}phenolato)dinickel(II) chloride

**DOI:** 10.1107/S160053681001442X

**Published:** 2010-04-24

**Authors:** Edward R. T. Tiekink, James L. Wardell, Christiane Fernandes, Adolfo Horn, Janet M. S. Skakle

**Affiliations:** a Department of Chemistry, University of Malaya, 50603 Kuala Lumpur, Malaysia; b Centro de Desenvolvimento Tecnológico em Saúde (CDTS), Fundação Oswaldo Cruz (FIOCRUZ), Casa Amarela, Campus de Manguinhos, Av. Brasil 4365, 21040-900, Rio de Janeiro, RJ, Brazil; c Laboratório de Ciências Químicas, Universidade Estadual do Norte Fluminense, 28013-602 Campos dos Goytacazes, RJ, Brazil; d Department of Chemistry, University of Aberdeen, Meston Walk, Old Aberdeen AB24 3UE, Scotland

## Abstract

The title salt, [Ni_2_(C_16_H_18_ClN_2_O_2_)_2_(CH_3_COO)]Cl, features a dinuclear cation in which the Ni atoms are triply bridged by two phenolate O ligands and a bidentate acetate ligand with all of the bridging distances essentially symmetric. Each Ni atom is also coordinated by the amine N, pyridine N and hydr­oxy O atoms of the 2-{[(3-chloro-2-hydroxy­prop­yl)(2-pyridylmeth­yl)amino]meth­yl}phenolate ligand which is, therefore, penta­dentate. The resultant N_3_O_3_ donor sets define octa­hedral coordination geometries. The chloride counter-anion is connected to the cation *via* two O_hydr­oxy_—H⋯Cl hydrogen bonds.

## Related literature

For the structure of the perchlorate salt, see: Horn *et al.* (2006 ▶). For the synthesis, see: Horn *et al.* (2000 ▶); Neves *et al.* (1993 ▶).
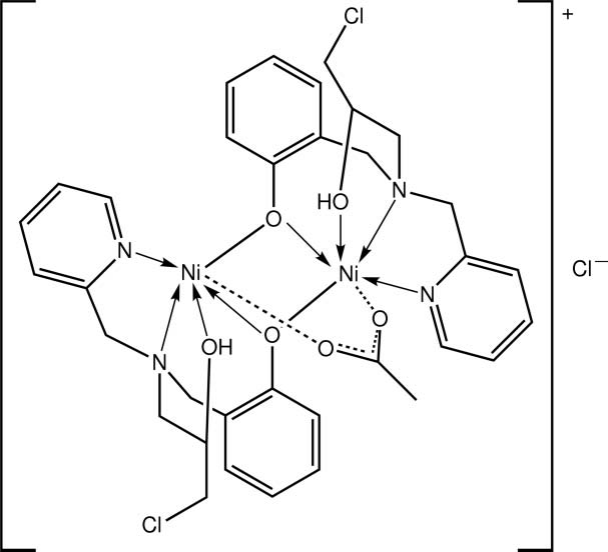


## Experimental

### 

#### Crystal data

[Ni_2_(C_16_H_18_ClN_2_O_2_)_2_(C_2_H_3_O_2_)]Cl
*M* *_r_* = 823.46
Orthorhombic, 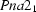

*a* = 19.0992 (6) Å
*b* = 18.0097 (5) Å
*c* = 10.1288 (3) Å
*V* = 3484.01 (18) Å^3^
*Z* = 4
Mo *K*α radiation
μ = 1.36 mm^−1^
*T* = 120 K
0.18 × 0.09 × 0.03 mm

#### Data collection

Nonius KappaCCD area-detector diffractometer
Absorption correction: multi-scan (*SADABS*; Sheldrick, 2007 ▶) *T*_min_ = 0.665, *T*_max_ = 1.000
25109 measured reflections
7859 independent reflections
6149 reflections with *I* > 2σ(*I*)
*R*_int_ = 0.070

#### Refinement

*R*[*F* ^2^ > 2σ(*F* ^2^)] = 0.049
*wR*(*F* ^2^) = 0.152
*S* = 1.05
7859 reflections
449 parameters
3 restraints
H-atom parameters constrained
Δρ_max_ = 0.82 e Å^−3^
Δρ_min_ = −0.75 e Å^−3^
Absolute structure: Flack (1983 ▶), 3653 Friedel pairs
Flack parameter: −0.012 (18)

### 

Data collection: *COLLECT* (Hooft, 1998 ▶); cell refinement: *DENZO* (Otwinowski & Minor, 1997 ▶) and *COLLECT*; data reduction: *DENZO* and *COLLECT*; program(s) used to solve structure: *SHELXS97* (Sheldrick, 2008 ▶); program(s) used to refine structure: *SHELXL97* (Sheldrick, 2008 ▶); molecular graphics: *ORTEP-3* (Farrugia, 1997 ▶) and *DIAMOND* (Brandenburg, 2006 ▶); software used to prepare material for publication: *publCIF* (Westrip, 2010 ▶).

## Supplementary Material

Crystal structure: contains datablocks global, I. DOI: 10.1107/S160053681001442X/zs2035sup1.cif

Structure factors: contains datablocks I. DOI: 10.1107/S160053681001442X/zs2035Isup2.hkl

Additional supplementary materials:  crystallographic information; 3D view; checkCIF report

## Figures and Tables

**Table 1:** Selected bond lengths (Å)

Ni1—O1	2.131 (4)
Ni1—O4	1.991 (4)
Ni1—O5	2.046 (4)
Ni1—O2	2.047 (4)
Ni1—N1	2.077 (5)
Ni1—N2	2.111 (4)
Ni2—O2	2.035 (4)
Ni2—O3	2.157 (4)
Ni2—O4	2.031 (4)
Ni2—O6	2.038 (4)
Ni2—N3	2.095 (5)
Ni2—N4	2.087 (4)

**Table 2:** Hydrogen-bond geometry (Å, °)

*D*—H⋯*A*	*D*—H	H⋯*A*	*D*⋯*A*	*D*—H⋯*A*
O1—H1o⋯Cl3	0.84	2.19	3.015 (4)	166
O3—H3o⋯Cl3	0.84	2.20	3.020 (4)	166

**Fig. 1.**
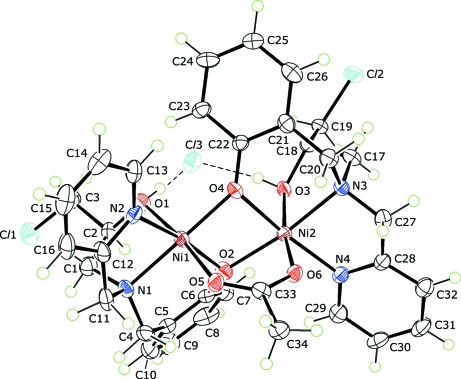
The molecular structure of the ionic components comprising (I) showing the atom-labelling scheme and displacement ellipsoids at the 50% probability level. The O–H···Cl hydrogen bonds are shown as black dashed lines.

## supplementary crystallographic information

### Comment

Complexes related to the title salt, (I), are of interest as synthetic models for urease (Horn *et al.*, 2006). The molecular structure of the dinuclear cation in (I) (Fig. 1) reveals the two Ni atoms to be triply bridged. Two of the bridges are provided by single O atoms derived from two phenoxides, and the third bridge arises from a bidentate acetate ligand with each of the bridging distances effectively symmetric (Table 1). Each Ni atom is also coordinated by the amino- and pyridine-N and hydroxy-O atoms so that the *N*-(2-oxybenzyl)-*N*-(2-pyridylmethyl)(3-chloro-2-hydroxy anion is pentadentate. Each Ni atom exists within similar octahedral N_2_O_4_ donor sets. The Cl^-^ anion is associated with the cation via two O–H···O hydrogen bonds (Fig. 1 and Table 2). The structure is isomorphous with the perchlorate salt (Horn *et al.*, 2006). The major difference between the structures of the cations relates to the disposition of the terminal chlorides. When each molecule is viewed down its respective Ni···Ni axis, both chlorides are orientated in the same direction for the perchlorate but, in (I), they are orientated in opposite directions.

### Experimental

The chloride salt was prepared analogously to the isostructural perchlorate salt (Horn *et al.*, 2006) from [Ni(OH_2_)_6_]Cl_2_ (1 mmol), *N*-(2-hydroxybenzyl)-*N*-(2-pyridylmethyl)-[(3-chloro)(2-hydroxy)]propylamine (1 mmol) (Neves *et al.*, 1993; Horn *et al.*, 2000), NaOAc (6 mmol) in MeOH. The crystals used in the structure determination were grown from MeOH solution. Anal. Found: C, 49.46; H, 4.85; N, 6.69%. Calc. for C_34_H_39_Cl_3_N_4_Ni_2_O_10_: C, 49.59; H, 4.77; N, 6.80%.

### Refinement

The O- and C-bound H atoms were geometrically placed (O–H = 0.84 Å and C–H = 0.95–1.00 Å) and refined as riding with *U_iso_*(H) = 1.2-1.5*U_eq_*(parent atom).

### Crystal data

**Table:**

[Ni_2_(C_16_H_18_ClN_2_O_2_)_2_(C_2_H_3_O_2_)]Cl	*F*(000) = 1704
*M**_r_* = 823.46	*D*_x_ = 1.570 Mg m^−^^3^
Orthorhombic, *P**n**a*2_1_	Mo *K*α radiation, λ = 0.71073 Å
Hall symbol: P 2c -2n	Cell parameters from 4394 reflections
*a* = 19.0992 (6) Å	θ = 2.9–27.5°
*b* = 18.0097 (5) Å	µ = 1.36 mm^−^^1^
*c* = 10.1288 (3) Å	*T* = 120 K
*V* = 3484.01 (18) Å^3^	Prism, light-blue
*Z* = 4	0.18 × 0.09 × 0.03 mm

### Data collection

**Table:**

Nonius KappaCCD area-detector diffractometer	7859 independent reflections
Radiation source: Enraf Nonius FR591 rotating anode	6149 reflections with *I* > 2σ(*I*)
10 cm confocal mirrors	*R*_int_ = 0.070
Detector resolution: 9.091 pixels mm^-1^	θ_max_ = 27.5°, θ_min_ = 2.9°
φ and ω scans	*h* = −17→24
Absorption correction: multi-scan (*SADABS*; Sheldrick, 2007)	*k* = −23→23
*T*_min_ = 0.665, *T*_max_ = 1.000	*l* = −13→12
25109 measured reflections	

### Refinement

**Table:**

Refinement on *F*^2^	Secondary atom site location: difference Fourier map
Least-squares matrix: full	Hydrogen site location: inferred from neighbouring sites
*R*[*F*^2^ > 2σ(*F*^2^)] = 0.049	H-atom parameters constrained
*wR*(*F*^2^) = 0.152	*w* = 1/[σ^2^(*F*_o_^2^) + (0.0888*P*)^2^] where *P* = (*F*_o_^2^ + 2*F*_c_^2^)/3
*S* = 1.05	(Δ/σ)_max_ = 0.001
7859 reflections	Δρ_max_ = 0.82 e Å^−^^3^
449 parameters	Δρ_min_ = −0.75 e Å^−^^3^
3 restraints	Absolute structure: Flack (1983), 3653 Friedel pairs
Primary atom site location: structure-invariant direct methods	Flack parameter: −0.012 (18)

### Special details

**Table:**

Geometry. All s.u.'s (except the s.u. in the dihedral angle between two l.s. planes) are estimated using the full covariance matrix. The cell s.u.'s are taken into account individually in the estimation of s.u.'s in distances, angles and torsion angles; correlations between s.u.'s in cell parameters are only used when they are defined by crystal symmetry. An approximate (isotropic) treatment of cell s.u.'s is used for estimating s.u.'s involving l.s. planes.
Refinement. Refinement of *F*^2^ against ALL reflections. The weighted *R*-factor wR and goodness of fit *S* are based on *F*^2^, conventional *R*-factors *R* are based on *F*, with *F* set to zero for negative *F*^2^. The threshold expression of *F*^2^ > 2σ(*F*^2^) is used only for calculating *R*-factors(gt) etc. and is not relevant to the choice of reflections for refinement. *R*-factors based on *F*^2^ are statistically about twice as large as those based on *F*, and *R*- factors based on ALL data will be even larger.

### Fractional atomic coordinates and isotropic or equivalent isotropic displacement parameters (Å^2^)

**Table:**

	*x*	*y*	*z*	*U*_iso_*/*U*_eq_	
Ni1	0.19933 (4)	0.51824 (3)	0.33432 (8)	0.02004 (16)	
Ni2	0.15818 (3)	0.66526 (3)	0.21542 (8)	0.01972 (16)	
Cl1	0.18142 (8)	0.72214 (8)	−0.33520 (14)	0.0302 (3)	
Cl2	0.47811 (8)	0.40832 (8)	0.34445 (17)	0.0359 (3)	
O1	0.2907 (2)	0.48411 (19)	0.2298 (4)	0.0247 (8)	
H1O	0.2991	0.5115	0.1646	0.037*	
O2	0.23231 (19)	0.62629 (19)	0.3420 (4)	0.0251 (8)	
O3	0.2253 (2)	0.6853 (2)	0.0480 (4)	0.0217 (8)	
H3O	0.2540	0.6509	0.0327	0.033*	
O4	0.1534 (2)	0.5554 (2)	0.1708 (4)	0.0234 (8)	
O5	0.1173 (2)	0.5475 (2)	0.4535 (4)	0.0244 (8)	
O6	0.0829 (2)	0.65273 (19)	0.3564 (4)	0.0225 (8)	
N1	0.2610 (2)	0.4867 (2)	0.4937 (4)	0.0226 (10)	
N2	0.1629 (2)	0.4082 (2)	0.3550 (5)	0.0218 (10)	
N3	0.0824 (2)	0.6913 (2)	0.0732 (4)	0.0196 (9)	
N4	0.1451 (2)	0.7777 (2)	0.2582 (5)	0.0231 (10)	
C1	0.3240 (3)	0.4468 (3)	0.4455 (6)	0.0270 (13)	
H1A	0.3127	0.3934	0.4361	0.032*	
H1B	0.3617	0.4514	0.5119	0.032*	
C2	0.3504 (3)	0.4764 (3)	0.3130 (6)	0.0239 (12)	
H2	0.3736	0.5257	0.3256	0.029*	
C3	0.4012 (3)	0.4222 (3)	0.2458 (6)	0.0320 (14)	
H3A	0.4148	0.4420	0.1584	0.038*	
H3B	0.3774	0.3739	0.2318	0.038*	
C4	0.2764 (3)	0.5511 (3)	0.5770 (6)	0.0288 (13)	
H4A	0.3014	0.5339	0.6570	0.035*	
H4B	0.2317	0.5738	0.6057	0.035*	
C5	0.3209 (3)	0.6105 (3)	0.5081 (6)	0.0271 (12)	
C6	0.2931 (3)	0.6461 (3)	0.3954 (5)	0.0235 (12)	
C7	0.3340 (3)	0.7018 (3)	0.3374 (6)	0.0288 (12)	
H7	0.3172	0.7266	0.2608	0.035*	
C8	0.3981 (3)	0.7214 (4)	0.3894 (7)	0.0398 (16)	
H8	0.4244	0.7599	0.3488	0.048*	
C9	0.4248 (3)	0.6858 (4)	0.4998 (8)	0.0391 (16)	
H9	0.4693	0.6990	0.5344	0.047*	
C10	0.3850 (3)	0.6305 (3)	0.5586 (6)	0.0330 (14)	
H10	0.4023	0.6061	0.6350	0.040*	
C11	0.2153 (3)	0.4350 (3)	0.5682 (6)	0.0278 (13)	
H11A	0.1815	0.4640	0.6215	0.033*	
H11B	0.2443	0.4053	0.6297	0.033*	
C12	0.1756 (3)	0.3833 (3)	0.4780 (6)	0.0263 (13)	
C13	0.1253 (3)	0.3658 (3)	0.2734 (6)	0.0287 (13)	
H13	0.1148	0.3842	0.1877	0.034*	
C14	0.1011 (3)	0.2963 (3)	0.3088 (7)	0.0352 (16)	
H14	0.0749	0.2672	0.2481	0.042*	
C15	0.1152 (3)	0.2708 (3)	0.4304 (8)	0.0376 (16)	
H15	0.0996	0.2228	0.4562	0.045*	
C16	0.1522 (3)	0.3143 (3)	0.5174 (7)	0.0340 (15)	
H16	0.1617	0.2969	0.6041	0.041*	
C17	0.1171 (3)	0.7182 (3)	−0.0491 (5)	0.0261 (12)	
H17A	0.1251	0.7723	−0.0416	0.031*	
H17B	0.0855	0.7097	−0.1251	0.031*	
C18	0.1868 (3)	0.6799 (3)	−0.0760 (5)	0.0211 (11)	
H18	0.1793	0.6267	−0.1007	0.025*	
C19	0.2292 (3)	0.7196 (3)	−0.1813 (5)	0.0246 (12)	
H19A	0.2743	0.6936	−0.1945	0.030*	
H19B	0.2394	0.7709	−0.1520	0.030*	
C20	0.0372 (3)	0.6240 (3)	0.0516 (6)	0.0262 (12)	
H20A	0.0175	0.6083	0.1376	0.031*	
H20B	−0.0025	0.6380	−0.0061	0.031*	
C21	0.0744 (3)	0.5590 (3)	−0.0095 (6)	0.0235 (12)	
C22	0.1311 (3)	0.5269 (3)	0.0579 (5)	0.0196 (11)	
C23	0.1641 (3)	0.4645 (3)	0.0001 (6)	0.0249 (12)	
H23	0.2027	0.4420	0.0433	0.030*	
C24	0.1405 (3)	0.4360 (3)	−0.1187 (6)	0.0311 (14)	
H24	0.1625	0.3936	−0.1556	0.037*	
C25	0.0853 (3)	0.4688 (3)	−0.1839 (6)	0.0274 (13)	
H25	0.0698	0.4495	−0.2662	0.033*	
C26	0.0526 (3)	0.5294 (3)	−0.1292 (6)	0.0293 (13)	
H26	0.0143	0.5516	−0.1742	0.035*	
C27	0.0408 (3)	0.7512 (3)	0.1318 (6)	0.0246 (12)	
H27A	0.0044	0.7294	0.1897	0.030*	
H27B	0.0169	0.7791	0.0607	0.030*	
C28	0.0848 (3)	0.8032 (3)	0.2102 (6)	0.0227 (11)	
C29	0.1830 (3)	0.8205 (3)	0.3388 (7)	0.0275 (12)	
H29	0.2255	0.8016	0.3741	0.033*	
C30	0.1619 (3)	0.8923 (3)	0.3728 (6)	0.0313 (14)	
H30	0.1894	0.9215	0.4312	0.038*	
C31	0.1006 (3)	0.9203 (3)	0.3207 (7)	0.0339 (14)	
H31	0.0862	0.9696	0.3405	0.041*	
C32	0.0604 (3)	0.8755 (3)	0.2388 (6)	0.0296 (13)	
H32	0.0176	0.8931	0.2028	0.036*	
C33	0.0792 (3)	0.6026 (3)	0.4437 (5)	0.0233 (12)	
C34	0.0251 (3)	0.6152 (3)	0.5522 (6)	0.0262 (12)	
H34A	−0.0032	0.5702	0.5633	0.039*	
H34B	−0.0053	0.6568	0.5278	0.039*	
H34C	0.0491	0.6266	0.6353	0.039*	
Cl3	0.34784 (8)	0.58123 (8)	0.01265 (16)	0.0320 (3)	

### Atomic displacement parameters (Å^2^)

**Table:**

	*U*^11^	*U*^22^	*U*^33^	*U*^12^	*U*^13^	*U*^23^
Ni1	0.0222 (4)	0.0200 (3)	0.0179 (3)	0.0015 (3)	−0.0005 (3)	0.0029 (3)
Ni2	0.0234 (4)	0.0184 (3)	0.0173 (3)	0.0009 (3)	0.0004 (3)	0.0020 (3)
Cl1	0.0378 (9)	0.0341 (7)	0.0187 (6)	0.0001 (6)	−0.0009 (6)	0.0014 (6)
Cl2	0.0244 (7)	0.0414 (7)	0.0419 (8)	0.0061 (6)	−0.0009 (7)	0.0031 (7)
O1	0.023 (2)	0.029 (2)	0.022 (2)	0.0013 (16)	−0.0012 (17)	0.0024 (18)
O2	0.026 (2)	0.0247 (17)	0.0252 (19)	−0.0030 (15)	−0.0050 (19)	0.0030 (18)
O3	0.023 (2)	0.0243 (19)	0.0182 (18)	0.0054 (16)	0.0007 (16)	0.0041 (16)
O4	0.029 (2)	0.0234 (18)	0.0180 (18)	0.0029 (16)	−0.0045 (16)	0.0037 (16)
O5	0.032 (2)	0.0199 (18)	0.0220 (19)	0.0044 (17)	0.0044 (17)	0.0045 (16)
O6	0.029 (2)	0.0181 (16)	0.021 (2)	0.0036 (14)	0.0046 (16)	0.0032 (16)
N1	0.025 (3)	0.024 (2)	0.019 (2)	0.0015 (19)	0.0013 (19)	0.0064 (19)
N2	0.026 (3)	0.0138 (19)	0.026 (3)	0.0015 (17)	0.007 (2)	−0.0002 (19)
N3	0.023 (2)	0.018 (2)	0.017 (2)	−0.0008 (18)	0.0017 (18)	0.0049 (18)
N4	0.028 (3)	0.020 (2)	0.021 (2)	0.0013 (19)	0.0039 (19)	0.0021 (19)
C1	0.025 (3)	0.028 (3)	0.028 (3)	0.002 (2)	0.003 (3)	0.001 (3)
C2	0.021 (3)	0.021 (3)	0.030 (3)	0.002 (2)	−0.002 (2)	0.002 (2)
C3	0.031 (3)	0.032 (3)	0.034 (3)	0.004 (2)	−0.004 (3)	−0.007 (3)
C4	0.028 (3)	0.040 (3)	0.019 (3)	0.012 (3)	−0.001 (2)	−0.003 (3)
C5	0.034 (3)	0.024 (3)	0.023 (3)	0.005 (2)	−0.002 (3)	−0.006 (2)
C6	0.028 (3)	0.023 (3)	0.019 (3)	0.003 (2)	−0.003 (2)	−0.006 (2)
C7	0.030 (3)	0.033 (3)	0.024 (3)	0.000 (2)	0.000 (3)	0.000 (3)
C8	0.033 (4)	0.044 (4)	0.043 (4)	−0.012 (3)	0.012 (3)	−0.017 (3)
C9	0.028 (4)	0.038 (3)	0.051 (4)	−0.002 (3)	−0.011 (3)	−0.010 (3)
C10	0.031 (4)	0.033 (3)	0.035 (3)	0.003 (3)	−0.008 (3)	−0.002 (3)
C11	0.025 (3)	0.030 (3)	0.029 (3)	0.010 (2)	0.006 (2)	0.014 (3)
C12	0.031 (3)	0.024 (3)	0.023 (3)	0.000 (2)	0.004 (2)	0.011 (2)
C13	0.031 (3)	0.020 (3)	0.035 (3)	0.003 (2)	0.002 (3)	0.007 (3)
C14	0.028 (3)	0.025 (3)	0.053 (5)	−0.002 (2)	0.011 (3)	−0.007 (3)
C15	0.027 (4)	0.028 (3)	0.058 (4)	0.005 (3)	0.016 (3)	0.012 (3)
C16	0.038 (4)	0.027 (3)	0.038 (4)	0.009 (3)	0.011 (3)	0.016 (3)
C17	0.027 (3)	0.033 (3)	0.018 (3)	−0.001 (2)	−0.003 (2)	0.003 (2)
C18	0.028 (3)	0.022 (3)	0.014 (2)	0.001 (2)	0.000 (2)	0.001 (2)
C19	0.030 (3)	0.026 (3)	0.018 (3)	0.002 (2)	−0.004 (2)	0.000 (2)
C20	0.020 (3)	0.032 (3)	0.026 (3)	0.001 (2)	0.002 (2)	0.000 (3)
C21	0.025 (3)	0.022 (3)	0.024 (3)	−0.003 (2)	−0.001 (2)	0.006 (2)
C22	0.024 (3)	0.017 (2)	0.019 (2)	−0.002 (2)	0.001 (2)	0.000 (2)
C23	0.030 (3)	0.024 (3)	0.021 (3)	0.000 (2)	0.005 (2)	−0.003 (2)
C24	0.037 (4)	0.037 (3)	0.018 (3)	−0.003 (3)	0.004 (2)	−0.006 (3)
C25	0.031 (3)	0.029 (3)	0.022 (3)	−0.003 (2)	0.000 (3)	−0.005 (3)
C26	0.027 (3)	0.035 (3)	0.026 (3)	−0.007 (2)	−0.004 (2)	0.007 (2)
C27	0.020 (3)	0.027 (3)	0.027 (3)	0.002 (2)	0.004 (2)	0.006 (2)
C28	0.032 (3)	0.018 (2)	0.019 (2)	0.002 (2)	0.008 (3)	0.004 (2)
C29	0.031 (3)	0.024 (2)	0.028 (3)	−0.002 (2)	0.005 (3)	0.005 (3)
C30	0.043 (4)	0.022 (3)	0.029 (3)	−0.005 (2)	0.007 (3)	−0.008 (3)
C31	0.045 (4)	0.019 (3)	0.037 (3)	0.003 (2)	0.006 (3)	−0.007 (3)
C32	0.032 (3)	0.025 (3)	0.032 (3)	0.001 (2)	0.004 (3)	0.001 (3)
C33	0.017 (3)	0.032 (3)	0.020 (3)	−0.002 (2)	0.002 (2)	0.001 (2)
C34	0.027 (3)	0.028 (3)	0.023 (3)	−0.002 (2)	0.006 (2)	0.001 (2)
Cl3	0.0358 (8)	0.0266 (7)	0.0335 (8)	0.0047 (6)	0.0120 (7)	0.0045 (6)

### Geometric parameters (Å, °)

**Table:**

Ni1—O1	2.131 (4)	C9—C10	1.387 (9)
Ni1—O4	1.991 (4)	C9—H9	0.9500
Ni1—O5	2.046 (4)	C10—H10	0.9500
Ni1—O2	2.047 (4)	C11—C12	1.508 (9)
Ni1—N1	2.077 (5)	C11—H11A	0.9900
Ni1—N2	2.111 (4)	C11—H11B	0.9900
Ni2—O2	2.035 (4)	C12—C16	1.379 (8)
Ni2—O3	2.157 (4)	C13—C14	1.382 (8)
Ni2—O4	2.031 (4)	C13—H13	0.9500
Ni2—O6	2.038 (4)	C14—C15	1.342 (10)
Ni2—N3	2.095 (5)	C14—H14	0.9500
Ni2—N4	2.087 (4)	C15—C16	1.376 (10)
Cl1—C19	1.807 (6)	C15—H15	0.9500
Cl2—C3	1.795 (6)	C16—H16	0.9500
O1—C2	1.425 (7)	C17—C18	1.524 (8)
O1—H1O	0.8400	C17—H17A	0.9900
O2—C6	1.330 (7)	C17—H17B	0.9900
O3—C18	1.458 (7)	C18—C19	1.518 (8)
O3—H3O	0.8400	C18—H18	1.0000
O4—C22	1.323 (6)	C19—H19A	0.9900
O5—C33	1.234 (7)	C19—H19B	0.9900
O6—C33	1.267 (6)	C20—C21	1.502 (8)
N1—C4	1.463 (7)	C20—H20A	0.9900
N1—C11	1.484 (7)	C20—H20B	0.9900
N1—C1	1.485 (7)	C21—C26	1.389 (8)
N2—C13	1.335 (7)	C21—C22	1.405 (8)
N2—C12	1.347 (7)	C22—C23	1.414 (7)
N3—C27	1.465 (7)	C23—C24	1.384 (8)
N3—C17	1.487 (7)	C23—H23	0.9500
N3—C20	1.504 (7)	C24—C25	1.376 (9)
N4—C28	1.332 (7)	C24—H24	0.9500
N4—C29	1.335 (8)	C25—C26	1.375 (8)
C1—C2	1.530 (8)	C25—H25	0.9500
C1—H1A	0.9900	C26—H26	0.9500
C1—H1B	0.9900	C27—C28	1.487 (8)
C2—C3	1.534 (8)	C27—H27A	0.9900
C2—H2	1.0000	C27—H27B	0.9900
C3—H3A	0.9900	C28—C32	1.413 (7)
C3—H3B	0.9900	C29—C30	1.397 (8)
C4—C5	1.534 (8)	C29—H29	0.9500
C4—H4A	0.9900	C30—C31	1.379 (9)
C4—H4B	0.9900	C30—H30	0.9500
C5—C10	1.374 (9)	C31—C32	1.389 (9)
C5—C6	1.413 (8)	C31—H31	0.9500
C6—C7	1.401 (8)	C32—H32	0.9500
C7—C8	1.379 (9)	C33—C34	1.526 (8)
C7—H7	0.9500	C34—H34A	0.9800
C8—C9	1.386 (10)	C34—H34B	0.9800
C8—H8	0.9500	C34—H34C	0.9800
			
O4—Ni1—O5	93.83 (16)	C8—C9—H9	120.8
O4—Ni1—O2	81.22 (15)	C10—C9—H9	120.8
O5—Ni1—O2	88.20 (15)	C5—C10—C9	121.1 (6)
O4—Ni1—N1	171.32 (17)	C5—C10—H10	119.4
O5—Ni1—N1	92.62 (17)	C9—C10—H10	119.4
O2—Ni1—N1	93.20 (17)	N1—C11—C12	112.0 (5)
O4—Ni1—N2	104.66 (17)	N1—C11—H11A	109.2
O5—Ni1—N2	86.02 (16)	C12—C11—H11A	109.2
O2—Ni1—N2	172.01 (18)	N1—C11—H11B	109.2
N1—Ni1—N2	81.57 (18)	C12—C11—H11B	109.2
O4—Ni1—O1	92.56 (15)	H11A—C11—H11B	107.9
O5—Ni1—O1	173.60 (16)	N2—C12—C16	120.6 (6)
O2—Ni1—O1	92.36 (15)	N2—C12—C11	116.4 (5)
N1—Ni1—O1	80.98 (17)	C16—C12—C11	122.9 (5)
N2—Ni1—O1	92.77 (15)	N2—C13—C14	122.5 (6)
O4—Ni2—O2	80.56 (14)	N2—C13—H13	118.8
O4—Ni2—O6	90.93 (14)	C14—C13—H13	118.8
O2—Ni2—O6	90.64 (15)	C15—C14—C13	118.8 (6)
O4—Ni2—N4	170.50 (17)	C15—C14—H14	120.6
O2—Ni2—N4	106.69 (17)	C13—C14—H14	120.6
O6—Ni2—N4	82.97 (16)	C14—C15—C16	119.7 (6)
O4—Ni2—N3	91.90 (16)	C14—C15—H15	120.1
O2—Ni2—N3	172.29 (16)	C16—C15—H15	120.1
O6—Ni2—N3	91.11 (16)	C15—C16—C12	119.7 (6)
N4—Ni2—N3	80.98 (17)	C15—C16—H16	120.2
O4—Ni2—O3	90.83 (15)	C12—C16—H16	120.2
O2—Ni2—O3	98.00 (15)	N3—C17—C18	113.0 (4)
O6—Ni2—O3	171.36 (15)	N3—C17—H17A	109.0
N4—Ni2—O3	94.15 (16)	C18—C17—H17A	109.0
N3—Ni2—O3	80.38 (16)	N3—C17—H17B	109.0
C2—O1—Ni1	113.0 (3)	C18—C17—H17B	109.0
C2—O1—H1O	111.7	H17A—C17—H17B	107.8
Ni1—O1—H1O	112.2	O3—C18—C19	107.8 (4)
C6—O2—Ni2	140.5 (3)	O3—C18—C17	104.9 (4)
C6—O2—Ni1	122.6 (3)	C19—C18—C17	112.2 (5)
Ni2—O2—Ni1	95.16 (15)	O3—C18—H18	110.6
C18—O3—Ni2	111.5 (3)	C19—C18—H18	110.6
C18—O3—H3O	96.9	C17—C18—H18	110.6
Ni2—O3—H3O	114.1	C18—C19—Cl1	110.4 (4)
C22—O4—Ni1	136.9 (3)	C18—C19—H19A	109.6
C22—O4—Ni2	125.8 (3)	Cl1—C19—H19A	109.6
Ni1—O4—Ni2	97.03 (16)	C18—C19—H19B	109.6
C33—O5—Ni1	127.6 (3)	Cl1—C19—H19B	109.6
C33—O6—Ni2	127.4 (4)	H19A—C19—H19B	108.1
C4—N1—C11	108.8 (4)	C21—C20—N3	114.5 (5)
C4—N1—C1	114.2 (4)	C21—C20—H20A	108.6
C11—N1—C1	109.8 (4)	N3—C20—H20A	108.6
C4—N1—Ni1	110.3 (3)	C21—C20—H20B	108.6
C11—N1—Ni1	103.5 (3)	N3—C20—H20B	108.6
C1—N1—Ni1	109.6 (3)	H20A—C20—H20B	107.6
C13—N2—C12	118.7 (5)	C26—C21—C22	119.9 (5)
C13—N2—Ni1	130.7 (4)	C26—C21—C20	121.0 (5)
C12—N2—Ni1	110.2 (4)	C22—C21—C20	119.1 (5)
C27—N3—C17	109.8 (4)	O4—C22—C21	120.5 (5)
C27—N3—C20	109.9 (4)	O4—C22—C23	121.6 (5)
C17—N3—C20	113.4 (4)	C21—C22—C23	118.0 (5)
C27—N3—Ni2	105.1 (3)	C24—C23—C22	120.6 (6)
C17—N3—Ni2	109.7 (3)	C24—C23—H23	119.7
C20—N3—Ni2	108.5 (3)	C22—C23—H23	119.7
C28—N4—C29	119.6 (5)	C25—C24—C23	120.5 (6)
C28—N4—Ni2	111.2 (3)	C25—C24—H24	119.7
C29—N4—Ni2	128.5 (4)	C23—C24—H24	119.7
N1—C1—C2	112.8 (5)	C26—C25—C24	119.6 (6)
N1—C1—H1A	109.0	C26—C25—H25	120.2
C2—C1—H1A	109.0	C24—C25—H25	120.2
N1—C1—H1B	109.0	C25—C26—C21	121.4 (6)
C2—C1—H1B	109.0	C25—C26—H26	119.3
H1A—C1—H1B	107.8	C21—C26—H26	119.3
O1—C2—C1	106.7 (4)	N3—C27—C28	112.0 (5)
O1—C2—C3	107.8 (5)	N3—C27—H27A	109.2
C1—C2—C3	112.1 (5)	C28—C27—H27A	109.2
O1—C2—H2	110.0	N3—C27—H27B	109.2
C1—C2—H2	110.0	C28—C27—H27B	109.2
C3—C2—H2	110.0	H27A—C27—H27B	107.9
C2—C3—Cl2	111.0 (4)	N4—C28—C32	121.8 (5)
C2—C3—H3A	109.4	N4—C28—C27	117.8 (4)
Cl2—C3—H3A	109.4	C32—C28—C27	120.3 (5)
C2—C3—H3B	109.4	N4—C29—C30	121.9 (6)
Cl2—C3—H3B	109.4	N4—C29—H29	119.0
H3A—C3—H3B	108.0	C30—C29—H29	119.0
N1—C4—C5	113.7 (5)	C31—C30—C29	119.3 (6)
N1—C4—H4A	108.8	C31—C30—H30	120.4
C5—C4—H4A	108.8	C29—C30—H30	120.4
N1—C4—H4B	108.8	C30—C31—C32	119.0 (5)
C5—C4—H4B	108.8	C30—C31—H31	120.5
H4A—C4—H4B	107.7	C32—C31—H31	120.5
C10—C5—C6	121.1 (6)	C31—C32—C28	118.5 (6)
C10—C5—C4	120.5 (5)	C31—C32—H32	120.8
C6—C5—C4	118.4 (5)	C28—C32—H32	120.8
O2—C6—C7	120.5 (5)	O5—C33—O6	126.6 (5)
O2—C6—C5	122.4 (5)	O5—C33—C34	117.5 (5)
C7—C6—C5	117.0 (5)	O6—C33—C34	115.8 (5)
C8—C7—C6	121.2 (6)	C33—C34—H34A	109.5
C8—C7—H7	119.4	C33—C34—H34B	109.5
C6—C7—H7	119.4	H34A—C34—H34B	109.5
C7—C8—C9	121.1 (6)	C33—C34—H34C	109.5
C7—C8—H8	119.5	H34A—C34—H34C	109.5
C9—C8—H8	119.5	H34B—C34—H34C	109.5
C8—C9—C10	118.5 (6)		
			
O4—Ni1—O1—C2	159.3 (3)	O6—Ni2—N4—C28	−72.9 (4)
O5—Ni1—O1—C2	−16.9 (15)	N3—Ni2—N4—C28	19.3 (4)
O2—Ni1—O1—C2	78.0 (3)	O3—Ni2—N4—C28	98.9 (4)
N1—Ni1—O1—C2	−14.9 (3)	O4—Ni2—N4—C29	147.3 (9)
N2—Ni1—O1—C2	−95.9 (3)	O2—Ni2—N4—C29	8.4 (5)
O4—Ni2—O2—C6	145.6 (6)	O6—Ni2—N4—C29	96.9 (5)
O6—Ni2—O2—C6	−123.5 (6)	N3—Ni2—N4—C29	−170.8 (5)
N4—Ni2—O2—C6	−40.7 (6)	O3—Ni2—N4—C29	−91.3 (5)
N3—Ni2—O2—C6	133.4 (12)	C4—N1—C1—C2	−90.3 (6)
O3—Ni2—O2—C6	56.1 (6)	C11—N1—C1—C2	147.1 (5)
O4—Ni2—O2—Ni1	−18.17 (15)	Ni1—N1—C1—C2	34.0 (5)
O6—Ni2—O2—Ni1	72.67 (16)	Ni1—O1—C2—C1	35.5 (5)
N4—Ni2—O2—Ni1	155.52 (16)	Ni1—O1—C2—C3	156.1 (4)
N3—Ni2—O2—Ni1	−30.4 (13)	N1—C1—C2—O1	−46.0 (6)
O3—Ni2—O2—Ni1	−107.68 (16)	N1—C1—C2—C3	−163.8 (5)
O4—Ni1—O2—C6	−149.3 (4)	O1—C2—C3—Cl2	−179.5 (4)
O5—Ni1—O2—C6	116.5 (4)	C1—C2—C3—Cl2	−62.4 (6)
N1—Ni1—O2—C6	24.0 (4)	C11—N1—C4—C5	−177.5 (5)
N2—Ni1—O2—C6	72.9 (13)	C1—N1—C4—C5	59.4 (6)
O1—Ni1—O2—C6	−57.1 (4)	Ni1—N1—C4—C5	−64.6 (5)
O4—Ni1—O2—Ni2	18.52 (15)	N1—C4—C5—C10	−120.3 (6)
O5—Ni1—O2—Ni2	−75.64 (16)	N1—C4—C5—C6	62.4 (7)
N1—Ni1—O2—Ni2	−168.16 (17)	Ni2—O2—C6—C7	−19.9 (9)
N2—Ni1—O2—Ni2	−119.3 (12)	Ni1—O2—C6—C7	140.9 (4)
O1—Ni1—O2—Ni2	110.74 (16)	Ni2—O2—C6—C5	163.0 (4)
O4—Ni2—O3—C18	72.2 (3)	Ni1—O2—C6—C5	−36.2 (7)
O2—Ni2—O3—C18	152.8 (3)	C10—C5—C6—O2	177.8 (5)
O6—Ni2—O3—C18	−29.5 (11)	C4—C5—C6—O2	−4.9 (8)
N4—Ni2—O3—C18	−99.7 (3)	C10—C5—C6—C7	0.6 (8)
N3—Ni2—O3—C18	−19.6 (3)	C4—C5—C6—C7	177.9 (5)
O5—Ni1—O4—C22	−117.5 (5)	O2—C6—C7—C8	−178.0 (5)
O2—Ni1—O4—C22	154.9 (5)	C5—C6—C7—C8	−0.8 (9)
N1—Ni1—O4—C22	104.6 (12)	C6—C7—C8—C9	1.0 (10)
N2—Ni1—O4—C22	−30.7 (5)	C7—C8—C9—C10	−1.0 (10)
O1—Ni1—O4—C22	62.9 (5)	C6—C5—C10—C9	−0.7 (10)
O5—Ni1—O4—Ni2	68.96 (17)	C4—C5—C10—C9	−177.9 (6)
O2—Ni1—O4—Ni2	−18.62 (15)	C8—C9—C10—C5	0.8 (10)
N1—Ni1—O4—Ni2	−68.9 (11)	C4—N1—C11—C12	158.8 (5)
N2—Ni1—O4—Ni2	155.85 (15)	C1—N1—C11—C12	−75.4 (6)
O1—Ni1—O4—Ni2	−110.61 (16)	Ni1—N1—C11—C12	41.6 (5)
O2—Ni2—O4—C22	−155.8 (4)	C13—N2—C12—C16	2.1 (8)
O6—Ni2—O4—C22	113.8 (4)	Ni1—N2—C12—C16	175.7 (5)
N4—Ni2—O4—C22	63.8 (12)	C13—N2—C12—C11	−177.1 (5)
N3—Ni2—O4—C22	22.6 (4)	Ni1—N2—C12—C11	−3.4 (6)
O3—Ni2—O4—C22	−57.8 (4)	N1—C11—C12—N2	−26.8 (7)
O2—Ni2—O4—Ni1	18.77 (16)	N1—C11—C12—C16	154.1 (6)
O6—Ni2—O4—Ni1	−71.72 (17)	C12—N2—C13—C14	−2.3 (8)
N4—Ni2—O4—Ni1	−121.6 (10)	Ni1—N2—C13—C14	−174.4 (4)
N3—Ni2—O4—Ni1	−162.86 (17)	N2—C13—C14—C15	0.8 (9)
O3—Ni2—O4—Ni1	116.74 (16)	C13—C14—C15—C16	0.9 (9)
O4—Ni1—O5—C33	−36.2 (5)	C14—C15—C16—C12	−1.1 (9)
O2—Ni1—O5—C33	44.9 (5)	N2—C12—C16—C15	−0.4 (9)
N1—Ni1—O5—C33	138.0 (5)	C11—C12—C16—C15	178.7 (6)
N2—Ni1—O5—C33	−140.6 (5)	C27—N3—C17—C18	149.5 (5)
O1—Ni1—O5—C33	140.0 (12)	C20—N3—C17—C18	−87.0 (6)
O4—Ni2—O6—C33	46.0 (4)	Ni2—N3—C17—C18	34.5 (5)
O2—Ni2—O6—C33	−34.6 (5)	Ni2—O3—C18—C19	160.7 (3)
N4—Ni2—O6—C33	−141.3 (5)	Ni2—O3—C18—C17	41.0 (5)
N3—Ni2—O6—C33	137.9 (4)	N3—C17—C18—O3	−50.0 (6)
O3—Ni2—O6—C33	147.7 (9)	N3—C17—C18—C19	−166.7 (4)
O4—Ni1—N1—C4	73.6 (12)	O3—C18—C19—Cl1	−175.2 (3)
O5—Ni1—N1—C4	−64.4 (4)	C17—C18—C19—Cl1	−60.3 (5)
O2—Ni1—N1—C4	24.0 (4)	C27—N3—C20—C21	179.5 (5)
N2—Ni1—N1—C4	−150.0 (4)	C17—N3—C20—C21	56.1 (6)
O1—Ni1—N1—C4	115.9 (4)	Ni2—N3—C20—C21	−66.1 (5)
O4—Ni1—N1—C11	−170.1 (10)	N3—C20—C21—C26	−120.4 (6)
O5—Ni1—N1—C11	51.9 (3)	N3—C20—C21—C22	61.2 (7)
O2—Ni1—N1—C11	140.2 (3)	Ni1—O4—C22—C21	150.4 (4)
N2—Ni1—N1—C11	−33.7 (3)	Ni2—O4—C22—C21	−37.5 (7)
O1—Ni1—N1—C11	−127.9 (3)	Ni1—O4—C22—C23	−30.7 (8)
O4—Ni1—N1—C1	−53.0 (12)	Ni2—O4—C22—C23	141.3 (4)
O5—Ni1—N1—C1	169.0 (3)	C26—C21—C22—O4	178.7 (5)
O2—Ni1—N1—C1	−102.6 (3)	C20—C21—C22—O4	−2.9 (8)
N2—Ni1—N1—C1	83.4 (3)	C26—C21—C22—C23	−0.2 (8)
O1—Ni1—N1—C1	−10.7 (3)	C20—C21—C22—C23	178.2 (5)
O4—Ni1—N2—C13	8.2 (5)	O4—C22—C23—C24	−179.3 (5)
O5—Ni1—N2—C13	101.2 (5)	C21—C22—C23—C24	−0.4 (8)
O2—Ni1—N2—C13	144.9 (11)	C22—C23—C24—C25	1.1 (9)
N1—Ni1—N2—C13	−165.6 (5)	C23—C24—C25—C26	−1.1 (9)
O1—Ni1—N2—C13	−85.2 (5)	C24—C25—C26—C21	0.5 (9)
O4—Ni1—N2—C12	−164.4 (4)	C22—C21—C26—C25	0.2 (8)
O5—Ni1—N2—C12	−71.5 (4)	C20—C21—C26—C25	−178.2 (5)
O2—Ni1—N2—C12	−27.7 (14)	C17—N3—C27—C28	−80.8 (5)
N1—Ni1—N2—C12	21.8 (4)	C20—N3—C27—C28	153.7 (4)
O1—Ni1—N2—C12	102.2 (4)	Ni2—N3—C27—C28	37.1 (5)
O4—Ni2—N3—C27	143.3 (3)	C29—N4—C28—C32	2.1 (8)
O2—Ni2—N3—C27	155.4 (11)	Ni2—N4—C28—C32	172.9 (4)
O6—Ni2—N3—C27	52.4 (3)	C29—N4—C28—C27	−174.0 (5)
N4—Ni2—N3—C27	−30.3 (3)	Ni2—N4—C28—C27	−3.2 (6)
O3—Ni2—N3—C27	−126.1 (3)	N3—C27—C28—N4	−24.1 (7)
O4—Ni2—N3—C17	−98.6 (3)	N3—C27—C28—C32	159.8 (5)
O2—Ni2—N3—C17	−86.5 (13)	C28—N4—C29—C30	−1.3 (8)
O6—Ni2—N3—C17	170.4 (3)	Ni2—N4—C29—C30	−170.3 (4)
N4—Ni2—N3—C17	87.7 (3)	N4—C29—C30—C31	−0.8 (9)
O3—Ni2—N3—C17	−8.1 (3)	C29—C30—C31—C32	1.9 (9)
O4—Ni2—N3—C20	25.8 (3)	C30—C31—C32—C28	−1.2 (9)
O2—Ni2—N3—C20	37.9 (14)	N4—C28—C32—C31	−0.9 (8)
O6—Ni2—N3—C20	−65.2 (3)	C27—C28—C32—C31	175.1 (5)
N4—Ni2—N3—C20	−147.9 (4)	Ni1—O5—C33—O6	0.4 (9)
O3—Ni2—N3—C20	116.3 (3)	Ni1—O5—C33—C34	−175.1 (4)
O4—Ni2—N4—C28	−22.5 (12)	Ni2—O6—C33—O5	−6.8 (9)
O2—Ni2—N4—C28	−161.5 (4)	Ni2—O6—C33—C34	168.8 (3)

### Hydrogen-bond geometry (Å, °)

**Table:**

*D*—H···*A*	*D*—H	H···*A*	*D*···*A*	*D*—H···*A*
O1—H1o···Cl3	0.84	2.19	3.015 (4)	166
O3—H3o···Cl3	0.84	2.20	3.020 (4)	166
